# Toward a DNA Taxonomy of Alpine *Rhithrogena* (Ephemeroptera: Heptageniidae) Using a Mixed Yule-Coalescent Analysis of Mitochondrial and Nuclear DNA

**DOI:** 10.1371/journal.pone.0019728

**Published:** 2011-05-17

**Authors:** Laurent Vuataz, Michel Sartori, André Wagner, Michael T. Monaghan

**Affiliations:** 1 Musée Cantonal de Zoologie, Lausanne, Switzerland; 2 Department of Ecology and Evolution, University of Lausanne, Lausanne, Switzerland; 3 Leibniz-Institute of Freshwater Ecology and Inland Fisheries (IGB), Berlin, Germany; Institut de Biologia Evolutiva - Universitat Pompeu Fabra, Spain

## Abstract

Aquatic larvae of many *Rhithrogena* mayflies (Ephemeroptera) inhabit sensitive Alpine environments. A number of species are on the IUCN Red List and many recognized species have restricted distributions and are of conservation interest. Despite their ecological and conservation importance, ambiguous morphological differences among closely related species suggest that the current taxonomy may not accurately reflect the evolutionary diversity of the group. Here we examined the species status of nearly 50% of European *Rhithrogena* diversity using a widespread sampling scheme of Alpine species that included 22 type localities, general mixed Yule-coalescent (GMYC) model analysis of one standard mtDNA marker and one newly developed nDNA marker, and morphological identification where possible. Using sequences from 533 individuals from 144 sampling localities, we observed significant clustering of the mitochondrial (*cox1*) marker into 31 GMYC species. Twenty-one of these could be identified based on the presence of topotypes (expertly identified specimens from the species' type locality) or unambiguous morphology. These results strongly suggest the presence of both cryptic diversity and taxonomic oversplitting in *Rhithrogena*. Significant clustering was not detected with protein-coding nuclear PEPCK, although nine GMYC species were congruent with well supported terminal clusters of nDNA. Lack of greater congruence in the two data sets may be the result of incomplete sorting of ancestral polymorphism. Bayesian phylogenetic analyses of both gene regions recovered four of the six recognized *Rhithrogena* species groups in our samples as monophyletic. Future development of more nuclear markers would facilitate multi-locus analysis of unresolved, closely related species pairs. The DNA taxonomy developed here lays the groundwork for a future revision of the important but cryptic *Rhithrogena* genus in Europe.

## Introduction

The accurate delimitation of species is an essential step in evolutionary biology, ecology, and conservation research [1]–[3]. DNA sequence variation has been broadly exploited as a delimitation tool (e.g. [4]–[6]) and one recent advance is the development of the general mixed Yule-coalescent (GMYC) model for single-locus data [7], . The GMYC approach estimates species boundaries directly from branching rates in mixed population-phylogenetic trees without the need for any prior definition of populations or species. This makes it suitable for large-scale, multi-species studies of taxonomic groups for which few genetic markers are readily available. A growing number of studies have applied the GMYC method of species delimitation to bacteria [9], fungi [10], algae [11], rotifers [7], [12], springtails [13], insects [1], [8], [14]–[25], crustaceans [26]–[28], mollusks [29], [30], amphibians [31] and mammals [32]. Most studies have relied on a single locus, often mitochondrial DNA (mtDNA), for GMYC analysis. Because incomplete sorting and hybridization of lineages can lead to inconsistent patterns among loci, any estimate of population status would benefit from additional unlinked loci. While the few studies that compared mtDNA groups with ribosomal DNA (rDNA) genotypes found the two markers to be largely congruent (e.g. [14]), it remains to be determined whether nuclear DNA (nDNA) forms sequence clusters that can be statistically identified with a coalescent approach and that are comparable to species.

*Rhithrogena* Eaton, 1881 (Ephemeroptera, Heptageniidae) is one of the three most species-rich mayfly genera on Earth [33]. It is among the most diverse genera of mayflies in Europe [34] with 69 described species as of February 2011 (http://www.faunaeur.org). Thirty of these occur in the Alps, of which 15 are strict Alpine endemics. Larvae inhabit well oxygenated, fast-flowing streams and rivers [35], including glacial rivers characteristic of the Alpine region [36]. They are abundant and ecologically important members of stream benthic (bottom) communities and some species can exploit extremely cold and torrential habitats [37]. Many of these Alpine habitats are affected by climate change [38] and their fauna is sensitive to alterations to discharge and temperature [39], [40]. Six *Rhithrogena* species are included in the Red List of threatened animal species for Switzerland [41] and four species are listed for Germany [42]. Despite their ecological and conservation importance, the taxonomic status of many European *Rhithrogena* species is unclear. Different “species groups” are recognized based on larval and adult morphologies [43]–[45]. While the groups themselves are easy to distinguish, species-level identification of the aquatic larvae is challenging [35] and morphological characters in the adults are scarce and of poor diagnostic value [46]. A recent origin, rapid morphological adaptation, convergence, and phenotypic plasticity all may be the cause of this ambiguous taxonomy (e.g. [47]).

Here we use single-locus approaches to evaluate the status of *Rhithrogena* species and species groups using standard primers for one mtDNA marker (*cox1*) and newly developed primers for one nDNA marker (PEPCK). We focus on European Alpine species using a geographically broad sampling scheme and first evaluated the monophyly of the species groups in our samples using both gene markers. We then applied both single and multiple-threshold GMYC models to each data set, with the specific aim to assess the suitability of nDNA by examining its congruence with *cox1* GMYC species and morphological species. Notably, we used expertly identified individuals sampled from type localities (topotypes) where possible to associate the GMYC species with named *Rhithrogena* species. We report high levels of variation in the PEPCK gene fragment, and although the mixed model did not provide a better fit than a null coalescent, using both markers in combination with morphological data provided resolution for many species of *Rhithrogena* and constitutes an important advance in our understanding of this morphologically cryptic group.

## Materials and Methods

### 2.1 Sampling

**Figure 1:**
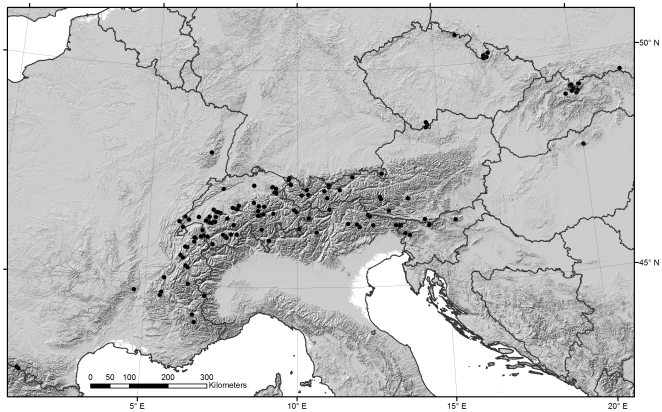
Sampling of European *Rhithrogena*. Filled circles represent the sampled localities.

Individuals were collected between September 2005 and March 2009 throughout the European Alps (France, Italy, Switzerland, Germany, Austria, Slovenia) and from additional localities in the Pyrenees and the Vosges Mountains (France), the Jura Mountains (France, Switzerland), the Tatra Mountains (Slovakia, Poland, Hungary), and the Bohemian Forest and the Sudete Mountains (Czech Republic; Fig. 1). We collected individuals from 22 type localities to increase confidence in assigning species names (Table 1). Larvae were collected in streams using Surber nets. Adutltts were caught using hand nets. All individuals were preserved in 100% ethanol in the field, returned to the laboratory, and stored at −20°C in fresh 100% ethanol.

**Table 1:** Sampled *Rhithrogena* species and populations.

Species groups	Species	Populations	*n*
alpestris	***allobrogica***	4	8
	*alpestris*	11	32
	***landai***	6	18
	*vaillanti*	6	17
diaphana	*beskidensis*	6	10
	***savoiensis***	6	16
hercynia	***corcontica***	1	3
	***gratianopolitana***	8	22
	***grischuna***	5	15
	*hercynia*	2	4
	spp	2	6
hybrida	***austriaca***	6	12
	***circumtatrica***	3	9
	***degrangei***	10	19
	***diensis***	2	6
	***endenensis***	8	19
	***hybrida***	8	20
	***mariaedominicae***	1	3
	***nivata***	4	12
	*puthzi*	5	12
	spp	9	22
loyolaea	*loyolaea*	14	36
	spp	8	21
semicolorata	*carpatoalpina*	9	27
	***colmarsensis***	6	14
	***dorieri***	6	14
	***fonticola***	2	6
	*germanica*	2	5
	***iridina***	2	6
	***picteti***	4	11
	***puytoraci***	6	8
	***rolandi***	2	6
	*semicolorata*	7	18
	***taurisca***	5	11
	spp	23	65

For each described species, the number of sampled populations (Populations) and individuals (*n*) are given within the corresponding species group. Individuals that could not be readily assigned to a described species are classed as “spp”. Species names in bold indicate that topotypes were sampled.

In the laboratory, individuals were first separated into the six species groups that occurred in our samples: alpestris, diaphana, hercynia, hybrida, loyolaea, and semicolorata. Where possible, individuals were identified to species according to the current morphological knowledge of the genus. Particular attention was paid to topotypes to ensure they fully corresponded to the published species description. Within four of the species groups there were a number of individuals that could not be attributed to a described species. These were designated “spp” within their respective species group (Table 1).

We selected 533 *Rhithrogena* individuals from 144 localities for genetic analysis. The number of individuals per morphologically identified species ranged from three (*Rh. mariaedominicae*; *Rh. corcontica*) to 36 (*Rh. loyolaea*), while the number of sampled localities ranged from one (*Rh. mariaedominicae*; *Rh. corcontica*) to 14 (*Rh. loyolaea*). Of the individuals assigned to *Rh.* spp within their respective species group, we sampled between six (hercynia group) and 65 (semicolorata group) individuals, corresponding to two and 23 localities. Three photographs (ventral, dorsal, and lateral view) of each individual were made using an Olympus ColorView IIIu camera (Olympus Corporation) connected to a Leica M205 C stereomicroscope (Leica Microsystems). As a result, a database of *ca.* 1600 photographs is available for later verification of morphological characters. In particular, we aimed to capture coloration that is lost using otherwise non-destructive DNA extraction (see next section).

### 2.2 PCR, sequencing and alignment

DNA was extracted using DNeasy Blood and Tissue kits (Qiagen, Hilden, Germany) as well as a BioSprint 96 extraction robot (Qiagen). Whole individuals were first soaked overnight in the extraction buffer with proteinase K at 56°C, leaving the gut and chitinous body parts intact. This treatment preserves the morphological characteristics of the individuals, which can be easily mounted for microscope identification. Extracted DNA, individuals and photographs are deposited at the Museum of Zoology, Lausanne, Switzerland. We amplified a 658-bp fragment of mitochondrial protein-coding cytochrome c oxidase subunit I (*cox1*), extensively used in species identification (e.g. DNA barcoding) and delimitation, using LCO1490 and HCO2198 primers [48]. We also amplified *ca.* 540 bp of nuclear protein-coding phosphoenolpyruvate carboxykinase (PEPCK) using newly designed primers Flv13 (5′-CTAACAGCACCAACCCCATT) and Rlv45 (5′-ACCTTGTGCTCKGCTGCT). Flv13 and the newly designed Rlv4 (5′-CTCATTGCTGCTCCAACAAA) PEPCK primer were used to amplify an individual of *Cinygmula* (Heptageniidae) as an outgroup. These PEPCK primers were designed from sequences first obtained using 19.5 dF and 22.5 drc primers for Lepidoptera [49]. Polymerase Chain Reaction (PCR) was conducted with a denaturation temperature of 94°C for 30 sec, an annealing temperature of 48°C for 30 sec (*cox1*) or ranging between 58°C and 62°C for 30 sec (PEPCK), and an elongation temperature of 72°C for one min for a total of 40 cycles, followed by a final extension for 10 min at 72°C.

All PCR products were visualized after agarose gel electrophoresis to verify amplicon size and detect possible contamination using negative controls. PCR products were purified using QIAquick PCR purification kits (Qiagen), and cycle-sequenced in both directions using BigDye v. 3.1 (Applied Biosystems, Foster City, CA). Sequences were analyzed using an ABI 3100 capillary sequencer (Applied Biosystems) at the Center for Integrative Genomics (CIG) at the University of Lausanne. Forward and reverse sequencing reads were assembled and edited using CodonCode Aligner v. 3.0.1 (CodonCode Corporation, Dedham, MA). The PEPCK heterozygous sites, typically identified as double peaks within the chromatograms, were coded according to the IUPAC code. Initial alignments were performed using ClustalW [50] as implemented in Jalview v. 2.4 [51]. Amino acid translation was then used to distinguish between coding (exon) and non-coding (intron) PEPCK regions, with intron boundaries identified using the GT-AG rule. A subsequent alignment of the PEPCK intron section was done using MAFFT v. 5 [52] in Jalview.

### 2.3 Best evolutionary models and partitioning schemes

We determined the best-fit evolutionary model for our complete data sets using MrAIC v. 1.4.3 [53]. A first attempt failed due to a large number of parameters compared with the sample size. To reduce the number of parameters while keeping the majority of sequence variation, we used reduced data sets for model determination. A single individual per population was randomly selected, resulting in 209 sequences for both *cox1* and PEPCK alignments. Identical *cox1* haplotypes and PEPCK genotypes were then removed from the alignments using Collapse 1.2 [54], with heterozygote and unknown bases considered as different characters from homozygous sites, resulting in final reduced alignments of 161 *cox1* haplotypes and 179 PEPCK genotypes (Table 2). A GTR+Γ+I and a JC69+Γ model were selected for the *cox1* and PEPCK reduced data sets, respectively, following the second-order Akaike information criterion (AICc) implemented in MrAIC, under the option using the models implemented in MrBayes [55]. In order to accommodate different substitution rates among codon positions, we used partitioned models of evolution (e.g. [56], [57]). Consequently, we examined *cox1* in two partitions, one with first and second codon positions and one with third positions (1+2, 3). For PEPCK, we used one partition with first and second codon positions and a second with third positions and the introns (1+2, 3+intron).

**Table 2:** Sequence variation measured within mitochondrial (*cox1*) and nuclear (PEPCK) gene regions of *Rhithrogena*.

	bp	*K*	*S*	*S*_i_	%*S*_i_
**reduced data set (** ***n*** ** = 209)**					
*cox1*	658	161	241	227	35
PEPCK	419	179	104	80	19
PEPCK_exons_	356	160	68	54	15
PEPCK_intron_	63	112	36	25	40
**complete data set (** ***n*** ** = 533)**					
*cox1*	658	312	243	232	35
PEPCK	419	390	117	95	23
PEPCK_exons_	356	339	79	63	18
PEPCK_intron_	63	164	38	32	51

Characteristics of the reduced data sets used to parameterize the model of evolution (top) and the complete data sets used for GMYC analysis (bottom) are specified. Also indicated are the relative contributions of coding (PEPCK_exons_) and non-coding (PEPCK_intron_) regions. *n* = number of sequences, bp = size of aligned data set, *K* = number of haplotypes (*cox1*) or genotypes (PEPCK), *S* = number of` polymorphic sites, *S*_i_ = number of parsimony-informative sites.

### 2.4 Phylogenetic analyses of species groups

For the phylogenetic analyses of *Rhithrogena* species groups, partitioned Bayesian inference searches were conducted separately for each gene using the reduced data sets under the selected models of evolution (see section 2.3 and Table 2). An individual of the related genus *Cinygmula* was used as an outgroup. Two independent analyses of four MCMC chains run for 10 million generations with a tree sampled each 1,000 generations were conducted for each gene using MrBayes v. 3.1.2, and performed at the freely available Bioportal (http://www.bioportal.uio.no). The stationary nucleotide frequencies and the alpha shape parameter of the gamma distribution (*cox1* and PEPCK), as well as the relative rate of substitution and the proportion of invariant sites (*cox1*), were unlinked across partitions. To allow the overall rates to vary across partitions, the ratepr command was set to variable (see [58]). One million (*cox1*) or two million (PEPCK) generations were discarded as a burnin after visually verifying that likelihood curves had flattened-out and that the independent runs converged using Tracer v. 1.4.1 [59].

### 2.5 Species delimitation using the GMYC model

The GMYC model combines equations that describe species branching events (macroevolution) and within-population coalescent branching (microevolution) on an ultrametric phylogenetic tree. The point of highest likelihood of this mixed model estimates the switch from speciation to coalescent branching and can be interpreted as the species boundary. A log-likelihood ratio test assesses if the mixed model fits the data significantly better than a null model that assumes a single coalescent process for the entire tree. In its original form [8], the GMYC model calculates a single transition across the entire tree. A more recent extension to the GMYC model allows for multiple lineages to each have their own transition threshold, where the single-threshold is used as a starting point and the threshold is then optimized one node toward the base of the tree and one node toward the terminals using an iteration process [19]. A log-likelihood ratio test assesses if the multiple model fits the data significantly better than the single model.

GMYC analyses were conducted independently on the complete *cox1* and PEPCK data sets. Ultrametric gene trees were reconstructed under a relaxed molecular clock (uncorrelated lognormal) model using BEAST v. 1.4.8 [59] at the Centre for High-Performance Computing of the Swiss Institute of Bioinformatics (http://www.vital-it.ch). Identical haplotypes (*cox1*) and genotypes (PEPCK) were first removed using Collapse as in section 2.3, resulting in matrices of 312 *cox1* haplotypes and 390 PEPCK genotypes (Table 2). The BEAST input files were generated using BEAUti v. 1.4.8 [59]. The evolutionary models and partitioning schemes as determined using the reduced data set (see section 2.3) were used, with the exception of the JC69 model for PEPCK, which is not implemented in BEAUti. In this case, we selected the best-fit model of evolution among those available in BEAUti following the AICc, which was the HKY+Γ model. The PEPCK partitioning scheme was then implemented by manually altering the input file. For both data sets, the mean substitution rate was set to one, the base frequencies were estimated from the data, and six gamma categories as well as a UPGMA starting tree were used. The substitution model, the rate heterogeneity and the base frequencies were unlinked across partitions. A coalescent (constant size) prior was preferred because a single coalescent cluster constitutes the GMYC null model (see [19]). All other parameters were set to default.

For both analyses, two independent MCMC chains were run for 50 million generations and sampled every 1,000 generations, resulting in 50,000 trees for each run. Run convergence was visually verified in Tracer as above. The first 5,000 trees were then discarded from each run and the independent log and tree files were combined using LogCombiner v. 1.4.8 [59], re-sampling one tree every 10 trees, resulting in 9,000 trees in the combined analyses of both data sets. All model parameters of the combined log files reached an estimated sample size (ESS)>200. The maximum clade credibility tree found using TreeAnnotator v. 1.4.8 [59] with all options set to default was used as input data for the GMYC model. Single and multiple-threshold GMYC models were optimized for each gene tree using the script available within the SPLITS package (available from http://r-forge.r-project.org/projects/splits/) for R.

### 2.6 Congruence of mitochondrial and nuclear DNA and morphology

A majority-rule consensus tree of PEPCK was built in MrBayes using the complete data set including the *Cinygmula* outgroup under the selected best-fit model of evolution and partitioning scheme (see section 2.3). This tree was then used to assess the congruence of cluster membership of individuals based on mitochondrial and nuclear markers in the absence of a significant fit of PEPCK to the GMYC model (see Results). Two independent analyses of four MCMC chains run for 10 million generations with a tree sampled each 1,000 generations were implemented and performed at Bioportal, and one million generations were removed from the analysis as a burnin. The stationary nucleotide frequencies and the alpha shape parameter of the gamma distribution were unlinked across partitions. Runs convergence was visually verified in Tracer as above.

Mitochondrial and nuclear markers were considered congruent when all individuals from one *cox1* GMYC species formed a unique PEPCK clade. To evaluate the congruence between mtDNA and morphological species of *Rhithrogena*, we first assigned names to *cox1* GMYC species when clusters contained all individuals from a single type locality. When no topotype was available, names were assigned to clusters if all individuals matched the species description. GMYC species that could not be linked to a name using either criterion above are referred to as “sp”. All sequences are available from GenBank (*cox1*: HM480851–HM481162 and JF423908; PEPCK: HM582943–HM583332 and JF423909). All matrices and trees are available from TreeBASE (http://purl.org/phylo/treebase/phylows/study/TB2:S11444).

## Results

### 3.1 PEPCK and *cox1* data

PEPCK fragments included a 173-bp exon, a length-variable intron (162–202 bp), and a second 183-bp exon. A number of individuals were heterozygous, with between one and 16 allelic polymorphisms detected in 305 (i.e. 57%) individuals, or a mean of 1.9 polymorphisms per individual in the entire matrix. Length-variation in 201 sequences (i.e. 38%) resulted in sequence chromatograms with 5′ or 3′ ends with continuous double peaks. When this occurred, forward and reverse chromatograms were edited separately and later merged into single sequences within the PEPCK alignment. A total of 63 bp within the intron could be unambiguously aligned (eight bp at the 5′ end; 55 bp at the 3′ end) and so were retained for analysis. The remaining intron region was not used for phylogenetic analysis. There were no insertions or deletions in the coding regions of the PEPCK fragment or in *cox1* sequences. The resulting PEPCK alignment length (419 bp) was shorter than *cox1* (658 bp), and although there were more PEPCK genotypes than *cox1* haplotypes, the number and proportion of parsimony-informative sites was higher for *cox1* (Table 2). Notably, nearly one-third of the total PEPCK variation occurred in the 63-bp intron region.

### 3.2 Species groups

**Figure 2:**
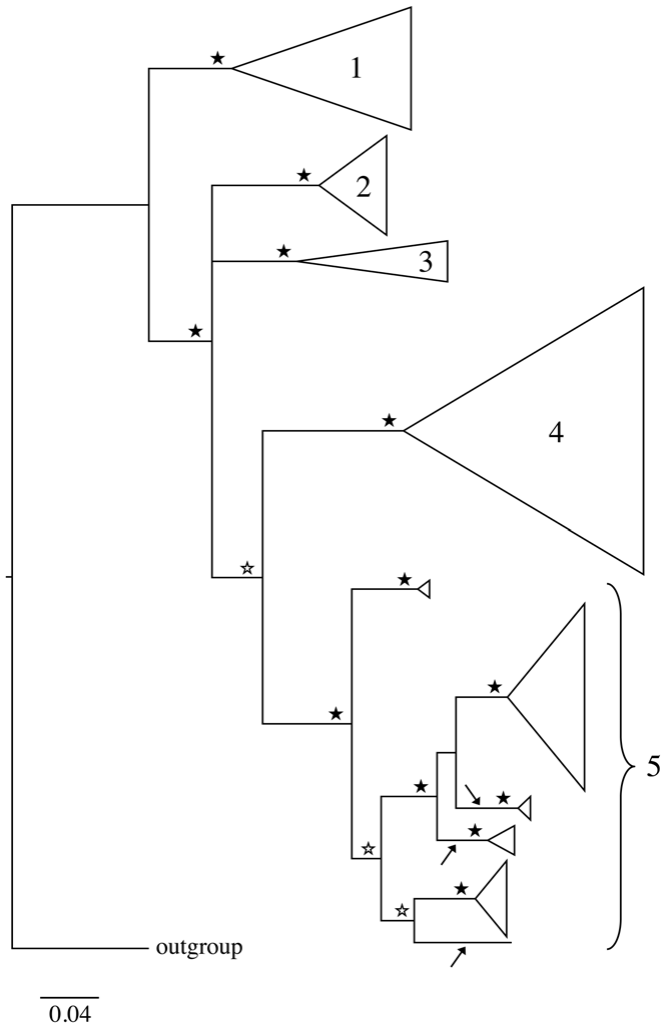
Bayesian majority-rule consensus tree of the reduced *cox1* data set obtained using MrBayes. Lineages 1 to 4 correspond to four different *Rhithrogena* morphological species groups (1: alpestris; 2: loyolaea; 3: diaphana; 4: semicolorata). Lineage 5 includes clades belonging to the hercynia species group (arrows) and the hybrida species group. Triangles represent collapsed lineages, (width proportional to the number of haplotypes). Filled stars indicate posterior probabilities (PP)>0.95, open stars indicate PP>0.75.

**Figure 3:**
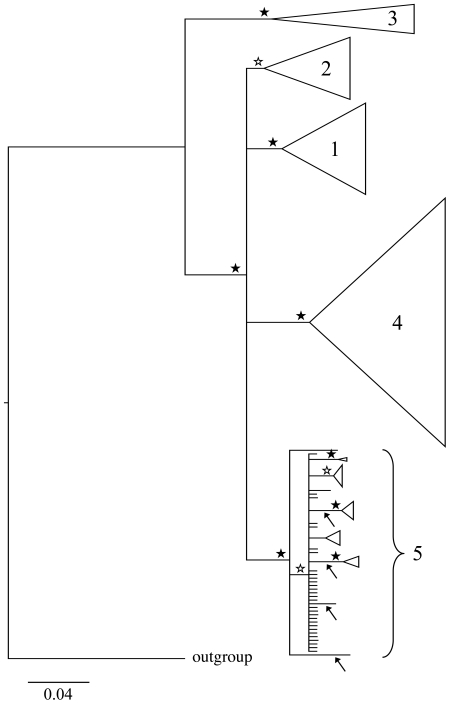
Bayesian majority-rule consensus tree of the reduced PEPCK data set obtained using MrBayes. Lineages 1 to 4 correspond to four different *Rhithrogena* morphological species groups (1: alpestris; 2: loyolaea; 3: diaphana; 4: semicolorata). Lineage 5 includes clades belonging to the hercynia species group (arrows) and the hybrida species group. Triangles represent collapsed lineages, (width proportional to the number of haplotypes). Filled stars indicate posterior probabilities (PP)>0.95, open stars indicate PP>0.75.

Four of the recognized species groups were recovered as monophyletic lineages with *cox1*: alpestris, loyolaea, diaphana, and semicolorata, each with posterior probabilities (PP) of 1.0 (Fig. 2). Neither the hybrida nor the hercynia species groups were monophyletic, but together formed a monophyletic lineage (PP = 1). This lineage is hereafter referred to as the hybrida species group. The same five monophyletic lineages were recovered in the analysis of PEPCK (Fig. 3). Clade support was equally high (PP = 1) except for the loyolaea group (PP = 0.85). One diaphana group individual (based on morphology and mtDNA) was recovered within the PEPCK hybrida lineage. However, the high number of missing (64 out of 419 bp) and heterozygous sites (16 versus a mean of 1.9 per sequence) within the PEPCK sequence of this individual possibly blurred the phylogenetic signal and may explain this incorrect attribution.

### 3.3 GMYC analysis

**Figure 4:**
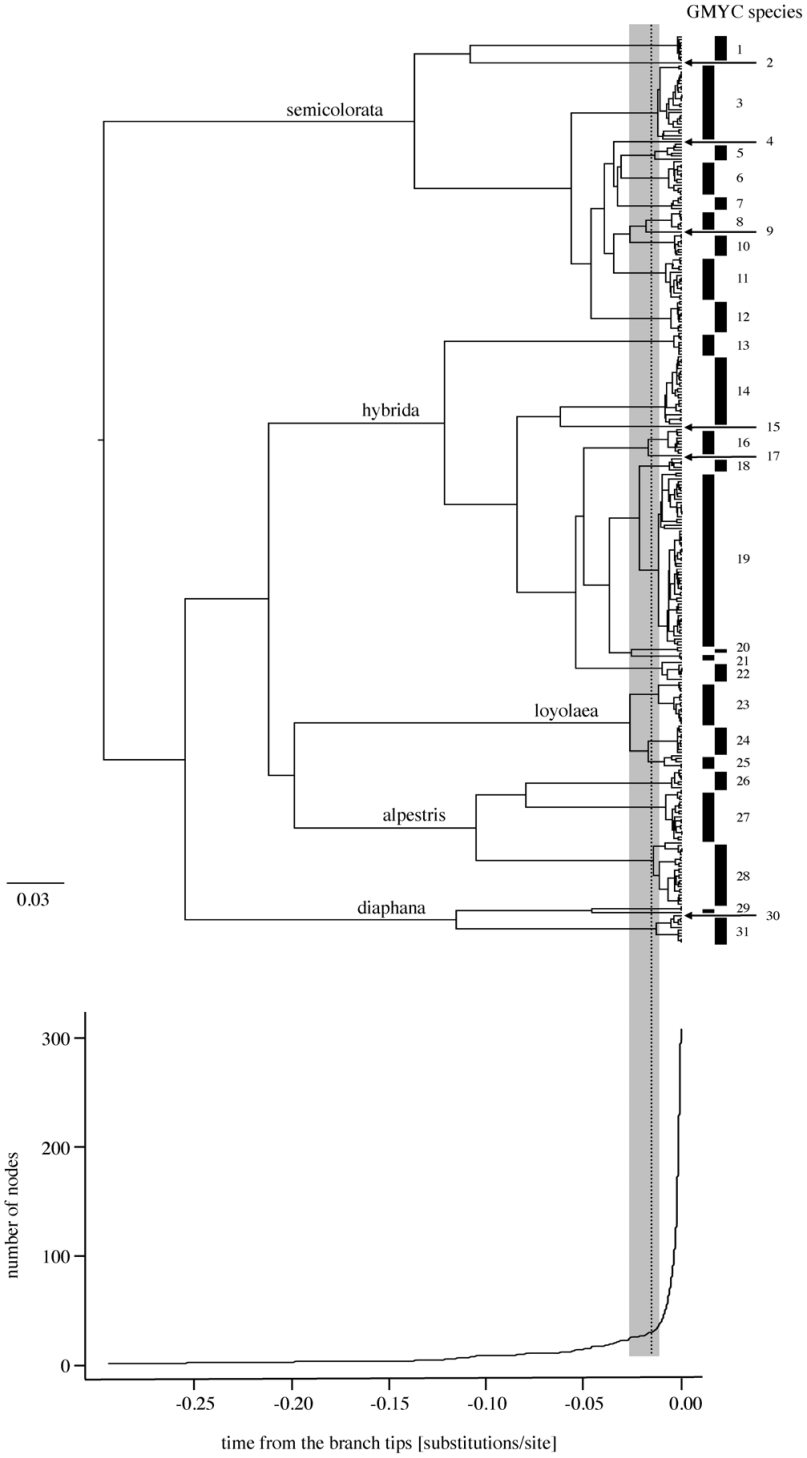
Clock-constrained Bayesian maximum clade credibility tree of the complete *cox1* data set obtained using BEAST. The tree (upper panel), obtained under a relaxed lognormal molecular clock, is presented with its corresponding lineage-through-time plot (lower panel). The broken vertical line indicates the point of maximum likelihood fit of the single-threshold GMYC model, i.e. the point of transition from interspecies (Yule) to intraspecies (coalescent) branching events. The grey shading corresponds to the confidence interval of the transition point. The bars indicate significant clusters (arrows: significant singletons) that are inferred to be species. The five species groups are specified on subtending branches. All GMYC clusters were well supported (PP≥0.99).

Both single and multiple-threshold GMYC models provided a better fit to the *cox1* ultrametric tree than the null model (likelihood ratio test, p<0.0005; Table 3). The single-threshold model delimited 31 putative species composed of 25 distinct clusters and six singletons (Fig. 4). This number corresponded well with the putative number of morphological species (Table 1). The multiple-threshold model delimited 80 putative species but did not fit the data significantly better that the single-threshold model (likelihood ratio test, p = 0.25). Neither single nor multiple-threshold GMYC models provided a better fit to the PEPCK ultrametric tree than the null model (p>0.05; Table 3).

**Table 3:** GMYC model outputs using single- and multiple-threshold approaches applied to *cox1* and PEPCK.

Data set	GMYC	L_o_	L_GMYC_	*T*	*N*_GMYC_	LR
*cox1*	single	2778.0	2798.0	0.0146	31	40.1***
	multiple		2803.7	-	80	51.5***
PEPCK	single	4122.6	4125.1	-	-	5.0 n.s.
	multiple		4129.0	-	-	12.9 n.s.

Likelihoods are indicated for null (L_o_) and GMYC (L_GMYC_) models, where null likelihoods are the same for single- and multiple-threshold models. *cox1* GMYC outputs include the threshold genetic distance from the branch tips where transition occurred (*T*, presented for single-threshold model), and the number of putative species as the sum of sequence clusters and singletons (*N*_GMYC_). Significance of the likelihood ratio (LR) was evaluated using a chi-square test (see section 2.5 for details of analyses).

*** = p<0.0005;

n.s. = not significant.

### 3.4 *cox1* GMYC species and congruence with nuclear DNA

Of the 31 putative species from the single-threshold GMYC analysis, 21 could be named. Eighteen of these were based on the occurrence of identified specimens from the type localities (topotypes) within the GMYC species cluster, and three were based on unambiguous identifications (Table 4). Single-threshold delimitation led to four cases of grouping and one case of splitting of recognized morphological species (Table 4). The most notable case of grouping was that of *Rh. austriaca*, *Rh. endenensis*, *Rh. hybrida* and *Rh. mariaedominicae* into a single GMYC species, all of which were sampled from the type localities. The other three cases of grouping involved two species each, all but one of which were also sampled from type localities. The remaining ten GMYC species could not be immediately linked to any described species according to our criteria of topotype or unambiguous identification of all members of the cluster (see section 2.6). The multiple-threshold result of 80 putative species led to a large number of splitting events, including splitting of topotype specimens. Based on the low congruence with morphological hypotheses and lack of significantly better fit than the single-threshold model (see above), we refer only to single-threshold results hereafter.

**Table 4:** *cox1* GMYC species congruence with PEPCK and morphology.

Species group	GMYC species	*Rhithrogena* species	PEPCK	Morphology
semicolorata	1	***dorieri***, ***colmarsensis***	+	
	2	*germanica*	+	+
	3	sp 1		
	4	sp 2		
	5	***taurisca***, ***rolandi***		
	6	***iridina***		+
	7	sp 3		
	8	***fonticola***		+
	9	sp 4		
	10	***picteti***		+
	11	***puytoraci***		+
	12	sp 5		
hybrida	13	***nivata***	+	+
	14	***degrangei***		+
	15	***grischuna***	+	+
	16	***gratianopolitana***		
	17	***gratianopolitana***		
	18	***circumtatrica***		+
	19	***austriaca***, ***endenensis***, ***hybrida***, ***mariaedominicae***		
	20	***diensis***		+
	21	sp 6		
	22	***corcontica***	+	+
loyolaea	23	sp 7		
	24	sp 8		
	25	sp 9		
alpestris	26	***allobrogica***	+	+
	27	***landai***, *vaillanti*	+	
	28	*alpestris*	+	+
diaphana	29	*beskidensis*		+
	30	sp 10		
	31	***savoiensis***	+	+

GMYC species are numbered as in Fig. 4 and species names are given when topotypes (bold) or unambiguous identifications were available. + = all individuals from one GMYC species formed an exclusive PEPCK clade (PEPCK column); + = GMYC species was associated to a single described species (Morphology column).

**Figure 5:**
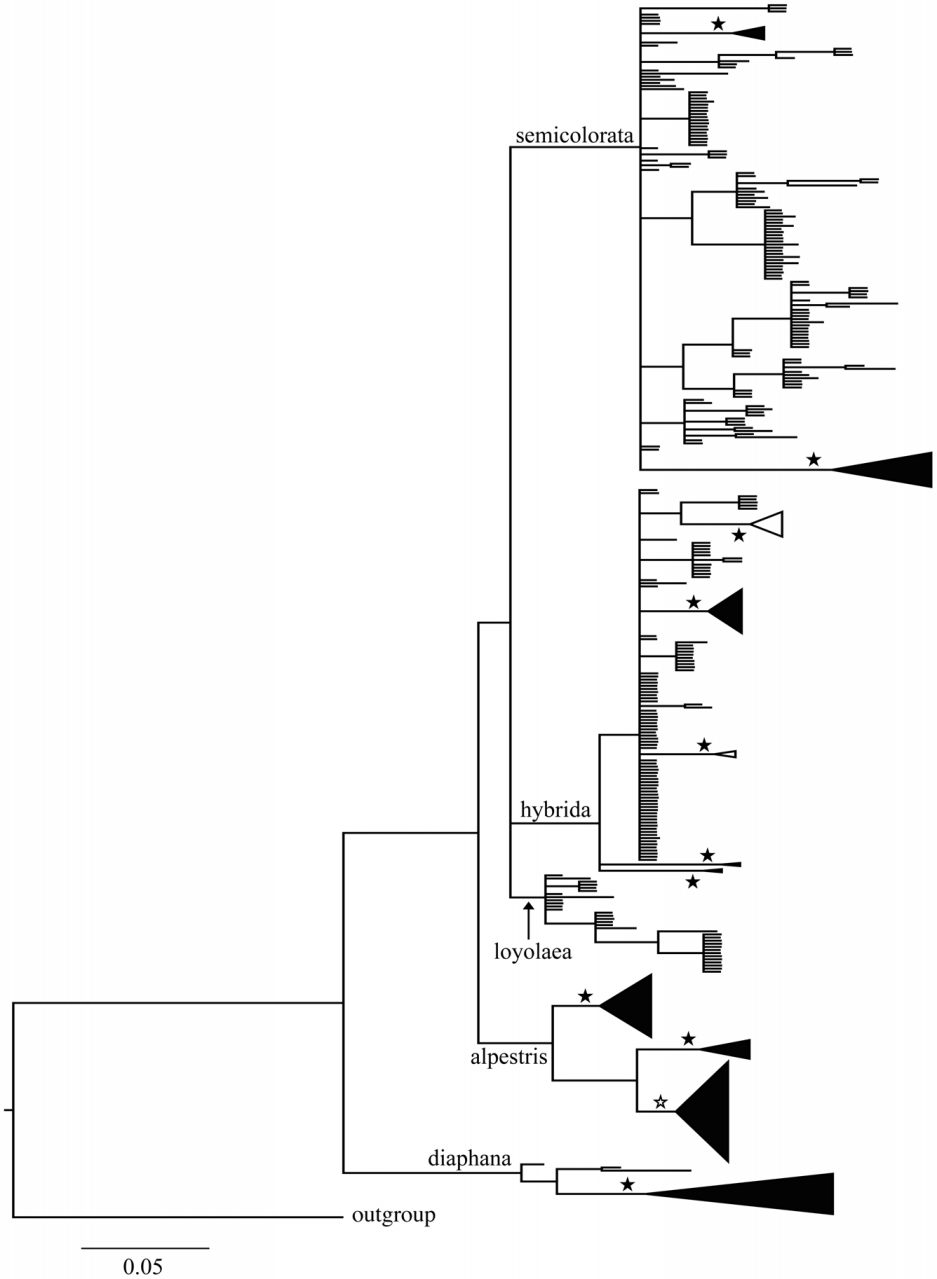
Bayesian majority-rule consensus tree of the complete PEPCK data set obtained using MrBayes. Triangles represent collapsed lineages, where filled triangles indicate clades congruent with *cox1* GMYC species (see Table 4) and open triangles indicate the clades containing a mixture of *Rh. gratianopolitana* individuals (GMYC species 16 and 17; see Fig. 4 and section 3.4). For each collapsed lineage, filled stars indicate PP values>0.95, open stars indicate PP>0.75. The five species groups are specified.

Nine GMYC species were fully congruent with PEPCK, in that all individuals of the GMYC species formed a unique, supported PEPCK clade (Fig. 5, Table 4). The single morphological species *Rh. gratianopolitana* was split into two GMYC species (GMYC 16 and 17) and these were incongruently distributed into two well supported (PP = 1) PEPCK clades (Fig. 4, 5). For the remaining 20 GMYC species, PEPCK variation was unresolved or incongruent. Overall, the degree of congruence varied by species group, with the highest degree of congruence found in the alpestris group (three out of three GMYC species), followed by the diaphana (33%), hybrida (30%), semicolorata (17%) and loyolaea groups (0%; Fig. 5; Table 4). Interestingly, the number of PEPCK genotypes was sometimes greater than the number of *cox1* haplotypes. Two GMYC species represented by a single mtDNA haplotype (i.e. “singletons” in the GMYC analysis) had five (*Rh. germanica*) and two (*Rh. grischuna*) PEPCK genotypes. In contrast, *Rh. nivata* had four times as many haplotypes as genotypes.

## Discussion

### 4.1 Species status of studied *Rhithrogena*

The aquatic larvae of many species of *Rhithrogena* mayflies inhabit sensitive Alpine environments. Several species are on the IUCN Red List, and many more recognized species only occur within small geographical areas and are of conservation interest. However, ambiguous morphological differences suggest that the current taxonomy may not accurately reflect the evolutionary diversity of the group. Our results strongly suggest that the current taxonomy of Alpine *Rhithrogena* results in the splitting of single evolutionary lineages. In several cases, individuals from more than one described specfident of the results, based on the fact that sequenced individuals came from type localities or were unambiguously identified. Such over-splitting may well lead to an overestimate of the degree of local endemism in the group, and probably results from the fact that morphological identification of *Rhithrogena* remains problematic [35], [46].

Our findings are somewhat in contrast to many studies using genetic methods that uncover large amounts of cryptic diversity (e.g. [12], [26], [27]), although the 10 GMYC species that remain unnamed in our study may include cryptic species. In this way, our findings are similar to other studies in which both splitting and lumping occurred [19], [30]. One consequence is that strictly using morphology to evaluate the congruence with genetic groups would have been overly subjective, and using *a priori* morphological species to calibrate a mean sequence divergence threshold (e.g. any barcoding paper) would have been largely meaningless. Our sampling scheme allowed us to use topotype samples to name clusters, and unambiguous identifications only when necessary.

Seven current species (*Rh. germanica*, *Rh. nivata*, *Rh. grischuna*, *Rh. corcontica*, *Rh. allobrogica*, *Rh. alpestris* and *Rh. savoiensis*) were confirmed by congruent nuclear and mitochondrial data. Two cases of mtDNA grouping (*Rh. dorieri*+*Rh. colmarsensis*; and *Rh. landai*+*vaillanti*) were also confirmed by PEPCK. For *Rh. dorieri*+*Rh. colmarsensis*, morphological discrimination is based solely on a small variation in the shape of the larval first gill plica [60], but two unlinked genetic markers both provide evidence for their being a single species. A further eight species (*Rh. iridina*, *Rh. fonticola*, *Rh. picteti*, *Rh. puytoraci*, *Rh. degrangei*, *Rh. circumtatrica*, *Rh. diensis and Rh. beskidensis*) were supported by mtDNA and topotype-morphology, although PEPCK data were not fully congruent. Our GMYC analysis also grouped *Rh. rolandi*+*Rh. taurisca*. These are thought to be differentiated by egg morphology [61], but a *Rh. rolandi* topotype shared the same haplotype with three *Rh. taurisca* topotypes. Moreover, their PEPCK genotypes are all included in the same non-exclusive clade, reducing the possibility of mtDNA hybridization and providing strong evidence that these represent a single species.

Future morphological study of mounted individuals and photographs may confirm or refute the mtDNA-based GMYC species assignation proposed in this study, as could sequencing additional gene fragments (see section 4.2). Hypotheses generated here include that *Rh. austriaca*, *Rh. endenensis*, *Rh. hybrida*, and *Rh. marieadominicae* together constitute a single species. *Rh. puthzi* could not be linked with confidence to any GMYC species due to the absence of a topotype or unambiguous identification. Nonetheless, all individuals tentatively identified as *Rh. puthzi* clustered with this group as well, suggesting that a total of five named species constitute this species. Four other species, *Rh. hercynia*, *Rh. loyolaea*, *Rh. carpatoalpina* and *Rh. semicolorata*, could not be readily assigned to clusters. The four individuals tentatively identified as *Rh. hercynia* clustered with *Rh. corcontica*, suggesting these could be synonymous. *Rh. loyolaea*, *Rh. carpatoalpina* and *Rh. semicolorata* certainly occur within our data set, but lacked topotype samples.

### 4.2 Nuclear genes in species-level studies

Most studies that use the GMYC approach to delineate species have relied on mtDNA, although evidence from unlinked loci is important for corroborating species status [4], [62]. The few studies that employed nuclear markers generally found a high degree of congruence with mtDNA (e.g. [14], [19]), but only Powell et al. [10] reported significant clustering in their analysis of 18S rDNA in fungi. Here we amplified and sequenced PEPCK for the first time in mayflies in order to provide a comparison with mtDNA in the absence of clear morphological differences in *Rhithrogena*. There was a high diversity of nDNA genotypes, but no species-coalescent transition could be detected and phylogenetic resolution was generally low. In the absence of significant clustering, we used a PEPCK phylogeny to assess congruence with mtDNA groups based on monophyly and clade support. This resulted in clear corroboration with approximately one-third of species. This low number resulted partly from a lack of phylogenetic signal. Introns contained many of the variable sites, which are potentially beneficial because of high substitution rates and conservative flanking regions [63]. Unfortunately, establishing sequence homology was problematic despite the fact that we studied closely related species here. For this reason, a number of variable sites were excluded when most of the intron was removed from our analysis. Even with a satisfactory alignment, models of sequence evolution are restricted to treating insertion/deletion events as binary characters (e.g. F81-like model in MrBayes), likely reducing the accuracy of branch-length estimates. Coding regions also had heterozygous positions, which also probably reduced the phylogenetic signal.

The incongruence in the two data sets most likely results from incomplete sorting of ancestral polymorphism in PEPCK. This is based on the presence of shared genotypes among what were otherwise independent species by morphology and mtDNA. Powerful phylogenetic methods have been developed to statistically account for incomplete sorting, but require multiple unlinked loci and thus have been applied only to relatively well characterized species or species pairs (e.g. [64], [65]). Here we studied more than 30 species belonging to multiple lineages within a genus. The advantage is that we gain a broad view of the extent of cryptic diversity and taxonomic oversplitting, and with future development of more nuclear markers, multi-locus approaches could be applied to unresolved, closely related species pairs.

### 4.3 Sampling effects on the GMYC

Sampling only a small number of populations may lead to artificial clustering within species when using the GMYC procedure [66], [67], although there are several lines of evidence to suggest this had no effect on our findings. We observed only one case of a morphological species being split into two (*Rh. gratianopolitana*) and this resulted from a single divergent mtDNA haplotype that was found at the same sampling locality as the rest of the cluster. In total, we observed four cases of genetic grouping of morphological species and can be fairly confident that this did not result from undersampling. Sampling within the Alps was extensive and there was little evidence for phylogenetic species (i.e. geographic isolation of unique haplotypes) in most of the 10 unidentified GMYC species. Three exceptions were (1) *Rh.* sp 4 (GMYC 9), which was the only semicolorata group individual from the Ardèche French department, and could potentially be either one of the closely related *Rh. picteti* (GMYC 10) or *Rh. fonticola* (GMYC 8); (2) *Rh.* sp 6 (GMYC 21) which comprised three individuals from one population. *Rh. diensis* (GMYC 20) was its closest *cox1* relative and was also composed of three individuals of a single population. These two GMYC species are separated by *ca.* 100 km of suitable habitat, and could potentially represent the same species; and (3) *Rh.* sp 10 (GMYC 30), which was a single individual from Switzerland. *Rh. beskidensis* was the closest *cox1* relative, sampled from the Bohemian Forest 400 kilometers away. Because the distribution of *Rh. beskidensis* includes the area from which GMYC 30 was sampled (http://www.faunaeur.org), these two GMYC species may be a single species.

### 4.4 Status of the *Rhithrogena* species groups

Our analysis recovered monophyletic alpestris, loyolaea, diaphana, and semicolorata species groups. The fifth well-supported lineage in our analysis contained individuals from both the hybrida and hercynia groups. The first attempt at grouping the European *Rhithrogena* species was undertaken by Jacob [43], who described six species groups (alpestris, dorieri, insularis, semicolorata, germanica, sowai). Sowa [44] described seven species groups (alpestris, hybrida, loyolaea, semicolorata, germanica, sowai, diaphana) using a new set of morphological characters. Using allozyme electrophoresis, Zurwerra et al. [45] recognized two groups (laevigata and lobata) that were each subdivided into two subgroups (semicolorata and diaphana for laevigata, hybrida and alpestris for lobata; [68]). Meanwhile, Sartori [34] proposed the hercynia group, closely related to the hybrida group, but characterized by the presence of dark spots on the upper face of the femora. Our results support the species groups as defined by Sowa [44], except that *Rh. germanica* was here within the semicolorata group rather than in the germanica group. An analysis including more members of the germanica group *sensu* Sowa [44] would help to determine whether this group should be fused with the semicolorata or whether it constitutes a monophyletic lineage, perhaps making semicolorata paraphyletic.
